# Classification and Interactions of LRR Receptors and Co-receptors Within the Arabidopsis Plasma Membrane – An Overview

**DOI:** 10.3389/fpls.2019.00472

**Published:** 2019-04-16

**Authors:** Lin Xi, Xu Na Wu, Max Gilbert, Waltraud X. Schulze

**Affiliations:** Department of Plant Systems Biology, University of Hohenheim, Stuttgart, Germany

**Keywords:** LRR-RKs, extracellular domain lengths, ligand-perceiving, co-receptor, plasma membrane

## Abstract

Receptor kinases (RK) constitute the largest protein kinase family in plants. In particular, members of the leucine-rich repeat-receptor kinases (LRR-RKs) are involved in the perception of various signals at the plasma membrane. Experimental evidence over the past years revealed a conserved activation mechanism through ligand-inducible heterodimer formation: a ligand is recognized by a receptor kinase with a large extracellular domain (ECD). This ligand binding receptor directly interacts with a so-called co-receptor with a small ECD for ligand fixation and kinase activation. A large proportion of LRR-RKs is functionally still uncharacterized and the dynamic complexity of the plasma membrane makes it difficult to precisely define receptor kinase heterodimer pairs and their functions. In this review, we give an overview of the current knowledge of LRR receptor and co-receptor functions. We use ECD lengths to classify the LRR receptor kinase family and describe different interaction properties of ligand-binding receptors and their respective co-receptor from a network perspective.

## Introduction

Receptor kinases (RK) form the largest phylogenetic kinase family in Arabidopsis (Shiu and Bleecker, 2003). The whole family of RK consists of over 600 members and represents nearly 2.5% of protein coding sequences in the Arabidopsis genome (Zulawski et al., 2014). Predictions suggest that around 400 family members are intrinsically located at the plasma membrane and some of these are well-studied cell surface RK (The Arabidopsis Genome Initiative, 2000; Shiu and Bleecker, 2001; Osakabe et al., 2013; Hohmann et al., 2017). Structurally, these RK consist of an extracellular region, a single membrane-spanning domain and an intracellular kinase domain. They function by recognizing various signals from the outside in response to environmental cues (Osakabe et al., 2013). Among these cell surface receptors, the subfamily containing leucine-rich repeats (LRR) in their extracellular domain (ECD) comprises the largest group with over 200 members (Diévart and Clark, 2003; Shinohara et al., 2016; Wu et al., 2016). The LRR tandem repeats and the phylogeny of kinase domain protein sequences define 14 subfamilies among the LRR-containing receptor kinases (LRR-RK; Shiu and Bleecker, 2001; Zulawski et al., 2014). Based on various experimental evidence, LRR-RKs can mainly be grouped into either regulating plant growth and development, or being involved in plant immunity and defence (He et al., 2018; Jamieson et al., 2018). However, for a majority of the LRR-RKs, the precise biological function is still uncharacterized.

Leucine-rich repeat -receptor kinases initiate cellular signaling cascades at the plasma membrane by perceiving ligands such as small polypeptides and/or hormones triggered by changing metabolism, environmental signals or infections (Ullrich and Schlessinger, 1990; He et al., 2000; Chinchilla et al., 2006; Yamaguchi et al., 2010; Endo et al., 2014; Matsubayashi, 2014; Zhang et al., 2016a). When a ligand is perceived by the ECD of the ligand-binding receptor, a heterodimer is formed with another LRR-RK, a so-called co-receptor, which usually has a short ECD (Nam and Li, 2002; Wang et al., 2008; Wang J. et al., 2015; Song et al., 2017; Perraki et al., 2018). At the same time, the cytoplasmic kinase domain of the ligand-perceiving receptor kinase is activated by autophosphorylation (Nemoto et al., 2011; Mitra et al., 2015). The kinase domain of the ligand-binding receptor through its output signal specificity also defines the signaling pathway to downstream biological processes. The shorter ECD of the co-receptor helps to hold the ligand and stabilizes and enhances transduction of the intracellular signal together with the ligand-binding receptor (Zhang et al., 2016a; Hohmann et al., 2017, 2018b). This activation mechanism is conserved across known LRR-RKs in plants (Fritz-Laylin et al., 2005; Aan den Toorn et al., 2015; Ma et al., 2016; Liu et al., 2017).

One of the structural hallmarks in LRR-RK signaling is the concept that ligand binding receptors have a larger ECD than co-receptors. RK with large ECDs of more than 12 LRR showed higher probability of heterotypic interactions with RK containing small ECDs of less than 12 LRR, compared to homotypic interactions of two receptors with small ECD, or two proteins with large ECD (Jaillais et al., 2011; Smakowska-Luzan et al., 2018). Recent structural analysis of interacting ECD heteromers suggested that the interaction between ECDs happens upon ligand binding, during which the ligand functions as a molecular glue between the two ECDs. As a result, during ligand binding, the larger ECD of the so-called ligand-binding receptor defines the specificity for the ligand, while the co-receptor is recruited by the ligand as a shape-complementary component (Santiago et al., 2013; Sun et al., 2013a, 2013b; Zhang et al., 2016a, 2016c). Initiation of different signaling pathways by heteromeric interactions of ligand binding LRR-RK and their respective co-receptors were especially well established by biochemical and genetics experiments on brassinosteroid receptor BRI1, phytosulfokine receptor PSKR1, the inflorescence deficient in abscission (IDA)-receptor HASEA involved in floral organ abscission, or the flagellin-receptor FLS2 (Nam and Li, 2002; Chinchilla et al., 2006; Albert and Felix, 2010; Mueller et al., 2012; Endo et al., 2014; Meng et al., 2015, 2016; Jorda et al., 2016; Song et al., 2016; Wang et al., 2016; Zhang et al., 2016c; Li et al., 2017; Hohmann et al., 2018b; Hu et al., 2018).

In this review, we performed classification of LRR-RKs into ligand-recognizing receptors and co-receptors based on their ECD sizes. Next, in view of published studies on receptor-co-receptor pairs, we generalized an interaction network of LRR-RKs to provide information on possible heterodimer pair preferences.

## Classification of LRR-RKs Based on Extracellular Domain Lengths

**FIGURE 1:**
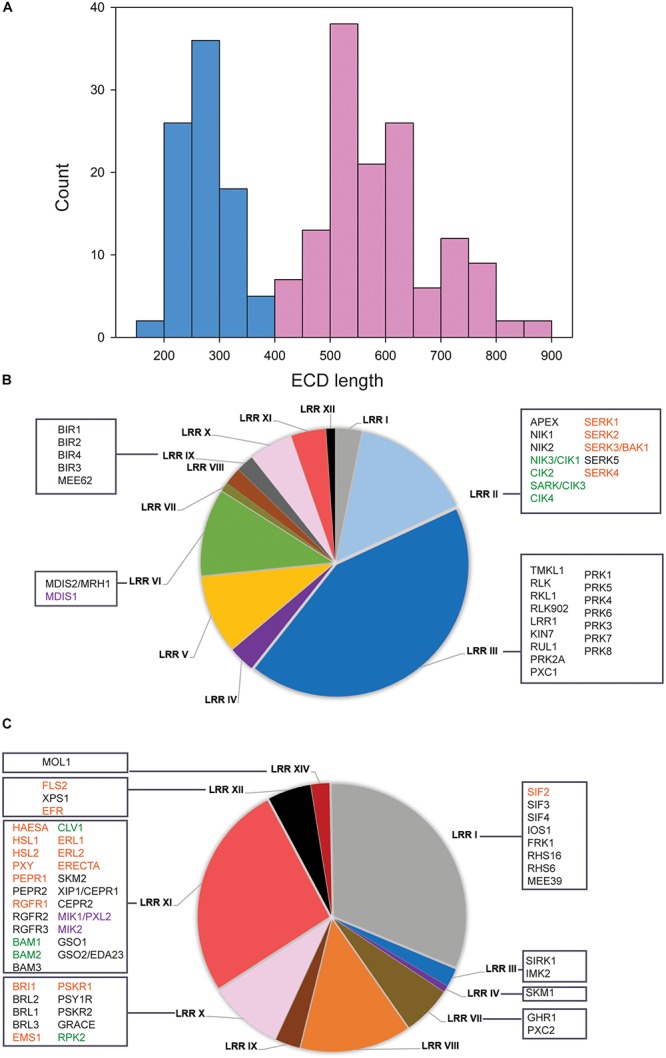
Classification of LRR-RKs by extracellular domain lengths. **(A)** Histogram of ECD lengths distribution. Light blue bars represent proteins with ECD shorter than 400 amino acids, in the following named “co-receptor group.” Light red bars represent proteins with ECD longer than 400 amino acids, in the following named “ligand-perceiving receptor group.” **(B)** Pie charts of subfamily composition in the “co-receptor group.” **(C)** Pie charts of subfamily composition in the “ligand-perceiving receptor group.” The square boxes contain gene symbols of LRR-RKs with known functions. Red font color: SERKs and their correspondence ligand binding receptors; Green font color: CIKs and their correspondence ligand binding receptors; Purple font color: MDIS1 and its interacting receptors MIKs.

It was recently proposed in several studies that ECD size could be used to predict LRR-RKs to function as receptors and co-receptors (Jaillais et al., 2011; Smakowska-Luzan et al., 2018). We followed this concept to systematically classify the 228 members of the LRR RK (Supplementary Table S1) into ligand binding receptor candidates and co-receptor candidates. A bimodal distribution of ECD lengths was observed with one maximum at around 250 amino acids and the second maximum of 550 amino acids (Figure 1A). Based on this distribution, we defined all protein with ECD length up to 400 amino acids as putative co-receptors, and those with a larger ECD greater than 400 amino acids were classified as putative ligand-recognition receptors (Figure 1A).

## Subfamilies and Functional Diversity of LRR-RKs

Within the “co-receptor group” about 60% of the proteins belonged to the LRR II and LRR III subfamily (Figure 1B), while the LRR I, LRR X, LRR XI and LRR XII subfamilies together constituted 68.4% within “ligand-perceiving receptor group” (Figure 1C). Systematic comparisons of tissue gene expression patterns across all LRR-RKs suggested that members from the same phylogenetic clade tended to have highly similar expression patterns (Wu et al., 2016). Therefore, the LRR-RKs from subfamilies LRR III and LRR X were suggested to share a higher degree of functional conservation, and this conclusion is supported by known functional redundancy of co-receptors such as somatic embryogenesis receptor kinases (SERKs) and CLAVATA3 insensitive receptor kinases (CIKs; Ma et al., 2016; Li et al., 2017; Cui et al., 2018; Hu et al., 2018).

To this date, SERKs and CIKs are the only members of the ‘co-receptor group’ with short ECD for which experimental evidence of their function as co-receptors is available (Figure 1B). SERKs have been found as co-receptor(s) in multiple pathways including brassinosteroid signaling (with receptor BRI1), immune responses (with receptors FLS2, PEPR1 and ERECTA), root meristem growth (with receptor RGF1), stomatal patterning (with receptor ERECTA), floral organ abscission (with receptor HAE and HSL2), vascular development (with receptor PXY1), anther cell fate definition (with receptor EMS1) and stress response (with receptor SIF2) (Nam and Li, 2002; Schulze et al., 2010; Roux et al., 2011; Meng et al., 2015, 2016; Song et al., 2016; Yeh et al., 2016; Zhang et al., 2016c; Li et al., 2017; Yuan et al., 2018). CIKs were recently shown to function in stem cell homeostasis (with receptor CLV1) and early anther development (with receptors RPK2, BAM1 and BAM2) (Cui et al., 2018; Hu et al., 2018). The corresponding ligand-binding receptors to these well-characterized co-receptors mostly come from the LRR X, LRR XI, and LRR XII subfamilies (Figure 1C). Other members of the LRRIII ‘co-receptors’ were functionally characterized, such as pollen receptor kinases (PRKs) with roles in pollen development, PXY/TDR-Correlated1 (PXC1) in secondary cell wall formation, RKL1 and RLK902 in root development, and reduced in lateral root growth 1 (RUL1) in cambium development (Tarutani et al., 2004; Agusti et al., 2011; Chang et al., 2013; Wang et al., 2013; Zhao et al., 2013; Yu et al., 2018). Although no precise functions for these proteins as co-receptors became evident, a structural study on pollen receptor kinases 3 (PRK3) reported its disulfide bonding pattern within the ECD similar to SERK co-receptors. This could indicate structural similarity between PRKs and SERKs in the folding of the ECD and thus could result in similar dimerization behavior (Dufayard et al., 2017; Chakraborty et al., 2018). Although up to now there is no direct functional evidence of disulfide-bonding patterns of co-receptors to be critical for heterodimerization, crystal structures of the ligand-binding receptor PXY1 revealed a structurally conserved ligand recognition mechanisms for different CLE peptides (Zhang et al., 2016b). Besides, PRK6 with its ECD C-terminal end has been proved to bind the ligand peptide LURE1, which is produced by the female tissue as an attractant for pollen tube guidance (Takeuchi and Higashiyama, 2016; Zhang et al., 2017).

The LRR VI subfamily occupied 11% of the “co-receptor group” (Figure 1B). Among these, putative co-receptor male discoverer 1 (MDIS1) was found to form heterodimers with receptors MDIS1-Interacting Receptor Kinases MIK1 and MIK2 in binding LURE1 (Wang et al., 2016). So far, LURE1 appears to be a ligand for multiple receptors (Cheung and Wu, 2016). Both short ECD receptors PRK6 and MDIS1 shows binding ability of LURE1. However, this precise mechanism is still unclear, especially since most members of the “co-receptor group” are functionally still uncharacterized (Wu et al., 2016). Thus, it is possible that more functional co-receptors could become evident in the near future. However, given the example of LURE1 to be recognized by two short ECD-domain receptors, it is likely that the “co-receptor group” might also contain unusual ligand-binding receptors.

**FIGURE 2:**
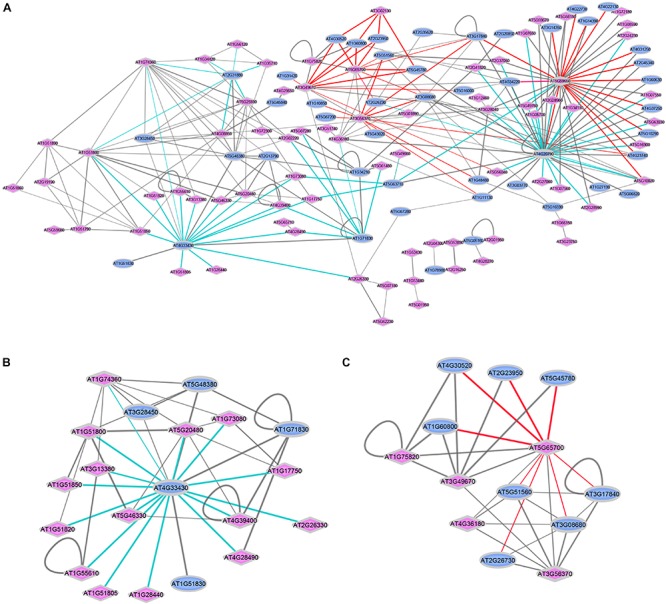
Interactions between ligand-perceiving receptors and co-receptors from published databases. **(A)** Network demonstration of 270 interaction events. Two interaction modes are marked with colored edges. Cyan edges: Mode-I, “co-receptor” is the center node surrounded by “ligand-perceiving receptors”; red edges: Mode-II, “ligand-perceiving receptor” is the center node surrounded by “co-receptors.” Blue ellipse: LRR-RKs from “co-receptor group”; Pink diamond: LRR-RKs from “ligand-perceiving group”. Width of edges: interaction values from STRING, MIND, TAIR and AI databases. **(B)** SERK3/BAK1 (AT4G33430) centered sub-network. **(C)** BAM1 (AT5G65700) centered sub-network.

Besides being involved as co-receptors in ligand binding, short ECD LRR-RKs seem to have additional functions during cell-surface signaling. For example, BAK1-interacting receptor kinases (BIRs) from the LRR IX subfamily within the “co-receptor group” were also shown to interact with different ligand-binding receptors. However, in contrast to signal enhancing co-receptors, binding of BIRs to the respective ligand-perceiving receptors inhibited or attenuated the signaling pathway (Imkampe et al., 2017; Hohmann et al., 2018a). As another example, the removal of APEX (LRR II subfamily) resulted in destabilization of several well-balanced LRR-RKs signaling pathways (Smakowska-Luzan et al., 2018). APEX is located in the center of our network, connecting to PEPR1 (AT1G73080) and PEPR2 (AT1G17750) (Figure 2A). Thus, LRR-RKs in the ‘co-receptor group’ generally play important roles during activation and stabilization, but also in fine-tuning of receptor complex functions at the plasma membrane.

## Dimerization Events and “Co-Receptor” Preference

The diversity of ligand-induced heterodimerization between different ligand-recognizing RK and their respective co-receptors raised the question of which dimer-pairs are preferentially formed, and whether specific interaction preference patterns can be distinguished. Thus, we extracted a total of 270 experimentally verified protein-protein interactions of LRR-RKs from public resources such as STRING (Szklarczyk et al., 2017), MIND (Jones et al., 2014), TAIR and AI (Swarbreck et al., 2008), and recent publications (Supplementary Table S2). We plotted these interaction pairs as a network with annotation as either “co-receptors” (blue ellipse) or “ligand-perceiving receptors” (pink diamond). Two general patterns could be distinguished (Figure 2A): Mode-I (cyan edges) describes one co-receptor interacting with different ligand-binding receptors, while Mode-II (red edges) describes a ligand-binding receptor interacting with different co-receptors.

Thus, Mode-I generalizes single co-receptors which can form complexes with different ligand-binding receptors. Thus these co-receptors can participate in different biological processes. The SERKs, and particularly SERK3/BAK1 (AT4G33430) are prominent examples of Mode-I co-receptors. In the isolated SERK3/BAK1 centered sub-network, there are 15 known ligand-perceiving receptors experimentally shown to form a functional signaling complex with SERK3/BAK1 (Figure 2B). These include well studied ligand-binding RK such as BRI1, FLS2, PSKR1 or HAESA. Mechanistically, when ligands are perceived by the ECDs of the respective ligand-perceiving receptors, the same co-receptor must be able to glue different ligands to the complex by using distinct recognition patches for each ligand. The short ECD of Mode-I co-receptor SERK3/BAK1, in fact, was shown feasible of stabilizing different ligand-receptor complexes through structurally different extracellular patches used with each of the different ligands (Hohmann et al., 2017). Based on the network view (Figure 2A), SERK1 (AT1G71830) and SOBIR1 (AT2G31880) are suggested to function as Mode-I co-receptors. The role of SOBIR1 in multiple signaling pathways is supported by recent preliminary findings that SOBIR1 can also participate in SERK1-HASEA pathway^1^ besides its known interaction with BAK1 to modulate receptor-like protein (RLP) signal transduction (Liebrand et al., 2014; Albert et al., 2015; van der Burgh et al., 2019).

Signal transition across the cell membrane is then mediated by the active kinase domain located at the intracellular part of the ligand-binding receptor. Chimeric fusion of extracellular and intracellular parts from different LRR-RKs suggests that the specificity of ligand perception is provided by the ECD of the ligand-binding receptor and its co-receptor, while the specific activation of cytoplasmic signaling cascades was suggested to be defined mainly by the output signal specificity of the intracellular kinase domain (He et al., 2000; Albert and Felix, 2010; Mueller et al., 2012; Hohmann et al., 2018b). However, recent evidence suggested a role of co-receptor phosphorylation also during specification of the signaling cascade. For example, phosphorylation at a characteristic tyrosine in the cytoplasmic domain of SERK3/BAK1 was shown to be important for ligand-induced activation of specific signaling pathways (e.g., immune signaling), but not in others (e.g., brassinosteroid signaling) (Perraki et al., 2018).

In Mode-II interactions (Figure 2C), the same ligand-binding receptor interacts with different co-receptors, as exemplified by the ligand-binding receptor barely any meristem1 (BAM1; AT5G65700) which was shown to form a complex with several co-receptors of the CIK-family (Cui et al., 2018). These multiple co-receptors belong to the same phylogenetic clade and share the high identity of both extracellular and intracellular domains (Shiu and Bleecker, 2001; Zhang et al., 2006; Zulawski et al., 2014; Ma et al., 2016). It will be exciting in the future to reveal the structural basis for the Mode-II interaction types, which also seems to involve RK BAM2 (AT3G49670), RKP2 (AT3G02130), AT3G56370, AT5G59650, and AT5G65700.

The network (Figure 2A) also illustrates known pairwise interactions of two co-receptors (Karlova et al., 2006) and heteromers of two ligand-perceiving receptors (Sun et al., 2012; Wang L. et al., 2015). Since co-receptors function in a broad way balancing ligand-binding receptor complex formation and their signaling, the interactions of two co-receptors may be important in titrating co-receptor availability for different signaling pathways. For example, BIR1 balances the amount of BAK1 available for receptor activation (Imkampe et al., 2017; Hohmann et al., 2018a). On the other hand, as the plasma membrane is highly organized and dynamic, co-receptors may also be involved in recruitment of ligand binding receptors and their substrates to specific membrane environments, such as membrane microdomains (Jones et al., 2014; Meng et al., 2015; Gao et al., 2018). The functional relevance of the heteromeric interaction of two ligand-binding receptors has to be elucidated.

## Conclusion and Perspective

Taken together, the available structural, genetic, and biochemical evidence has revealed conserved mechanisms in dimerization of ligand-binding receptors with their co-receptors. We confirmed the classification of LRR-containing receptor kinases as ligand-binding receptors and co-receptors based on ECD lengths and summarized distinct heteromeric interaction modes in a network view. This classification and the network view will help to predict the function of yet uncharacterized LRR-RKs within Mode-I or Mode-II interaction types. LRR-RKs classified within the “co-receptor group” were shown to have diverse functions in activation, stabilization and fine-tuning of ligand-binding receptors. Thus, co-receptors mediate signaling across the plasma membrane in the context of different receptor kinase complexes.

## Author Contributions

LX, XW, and WS collected the works of literature. MG gathered interaction information for LRR-RKs. LX and WS wrote the manuscript.

## Conflict of Interest Statement

The authors declare that the research was conducted in the absence of any commercial or financial relationships that could be construed as a potential conflict of interest.

## Footnotes

1 http://dx.doi.org/10.1101/453944

## Supplementary Material

TABLE S1 List of LRR-RKs used in this work.

TABLE S2 Interaction partners for the network construction.
